# Supplementary material for: High Prevalence of Three Potyviruses Infecting Cucurbits in Oklahoma and Phylogenetic Analysis of Cucurbit Aphid-Borne Yellows Virus Isolated from Pumpkins
Source: Pathogens. 2021 Jan 8;10(1):53. doi: 10.3390/pathogens10010053 (PMC7828045; doi:10.3390/pathogens10010053)
Supplement: Supplementary file 1 [file pathogens-10-00053-s001.zip › Supplymentary Tables S1-S2.revised.docx]

**Table S1**: List of viruses infecting cucurbits.

| No. | Family | Genus | Virus name | Abbreviation | Reference/s |
| --- | --- | --- | --- | --- | --- |
| 1 | *Alphaflexviridae* | *Potexvirus* | Alstroemeria virus x | AVX | 1 |
| 2 | *Betaflexiviridae* | *Carlavirus* | Cucumber vein clearing virus | CVCV | 2 |
| 3 | *Betaflexiviridae* | *Carlavirus* | Melon yellowing-associated virus | MYAV | 2 |
| 4 | *Betaflexiviridae* | *Carlavirus* | Muskmelon vein necrosis virus | MVNV | 2 |
| 5 | *Betaflexiviridae* | *Carlavirus* | White bryony mosaic virus | WBMV | 3 |
| 6 | *Betaflexiviridae* | *Ourmiavirus* | Ourmia melon virus | OMV | 4 |
| 7 | *Betaflexiviridae* | *Unassigned* | Watermelon virus A | WVA | 5 |
| 8 | *Bromoviridae* | *Alphamovirus* | Alfalfa mosaic virus | AaMV | 6 |
| 9 | *Bromoviridae* | *Cucumovirus* | Cucumber mosaic virus | CMV | 7 |
| 10 | *Bromoviridae* | *Ilavirus* | Apple mosaic virus | AMV | 8 |
| 11 | *Bromoviridae* | *Ilavirus* | Prune dwarf virus | PNV | 9 |
| 12 | *Bromoviridae* | *Ilavirus* | Prunus necrotic ringspot virus | PNRSV | 9 |
| 13 | *Bunyaviridae* | *Tospovirus* | Groundnut bud necrosis virus | GNBNV | 10 |
| 14 | *Bunyaviridae* | *Tospovirus* | Melon severe mosaic virus | MSMV | 11 |
| 15 | *Bunyaviridae* | *Tospovirus* | Zucchini lethal chlorosis virus | ZLCV | 12 |
| 16 | *Closteroviridae* | *Closterovirus* | Beet pseudo yellows virus | BPYV | 13 |
| 17 | *Closteroviridae* | *Crinivirus* | Cucumber yellows virus | CYV | 14 |
| 18 | *Closteroviridae* | *Crinivirus* | Cucurbit chlorotic yellow virus | CCYV | 15 |
| 19 | *Closteroviridae* | *Crinivirus* | Cucurbit yellow stunting disorder virus | CYSDV | 13 |
| 20 | *Closteroviridae* | *Crinivirus* | Lettuce infectious yellow virus | LIYV | 16 |
| 21 | *Edornaviridae* | *Edornavirus* | Cucumis melo edornavirus | CMEV | 17 |
| 22 | *Edornaviridae* | *Edornavirus* | Lageneria siceraria edornavirus-california | LSEV-Cal | 18 |
| 23 | *Edornaviridae* | *Edornavirus* | Lageneria siceraria edornavirus-Hubei | LSEV-Hub | 19 |
| 24 | *Geminiviridae* | *Begomovirus* | Cucurbit leaf crumple virus/cucurbit leaf curl virus | CLCV | 20,21 |
| 25 | *Geminiviridae* | *Begomovirus* | Bitter gourd yellow vein virus | BGYVV | 22 |
| 26 | *Geminiviridae* | *Begomovirus* | Chayote yellow mosaic virus | CYMV | 23 |
| 27 | *Geminiviridae* | *Begomovirus* | Luffa yellow mosaic virus | LYMV | 24 |
| 28 | *Geminiviridae* | *Begomovirus* | Melon chlorotic leaf curl virus | MCLCV | 25 |
| 29 | *Geminiviridae* | *Begomovirus* | Melon chlorotic mosaic virus | MCMV | 26 |
| 30 | *Geminiviridae* | *Begomovirus* | Pumpkin yellow vein mosaic virus | PYVMV | 27 |
| 31 | *Geminiviridae* | *Begomovirus* | Squash leaf curl china virus | SLCCV | 28 |
| 32 | *Geminiviridae* | *Begomovirus* | Squash leaf curl philippines virus | SLCPV | 29 |
| 33 | *Geminiviridae* | *Begomovirus* | Squash leaf curl virus | SLCV | 30 |
| 34 | *Geminiviridae* | *Begomovirus* | Squash leaf curl yunnan virus | SLCYV | 31 |
| 35 | *Geminiviridae* | *Begomovirus* | Squash mild leaf curl virus | SMLCV | 29 |
| 36 | *Geminiviridae* | *Begomovirus* | Squash yellow mild mottle virus | SYMMV | 32 |
| 37 | *Geminiviridae* | *Begomovirus* | Telfairia golden mosaic virus/Telfairia mosaic virus | TGMV | 33 |
| 38 | *Geminiviridae* | *Begomovirus* | Tomato leaf curl virus | TLCV | 3 |
| 39 | *Geminiviridae* | *Begomovirus* | Tomato leaf curl barbados virus | TLCBV | 34 |
| 40 | *Geminiviridae* | *Begomovirus* | Tomato leaf curl new delhi virus | TLCNDV | 35 |
| 41 | *Geminiviridae* | *Begomovirus* | Tomato yellow leaf curl virus | TYLCV | 36 |
| 42 | *Geminiviridae* | *Begomovirus* | Watermelon chlorotic stunt virus | WCSV | 37 |
| 43 | *Geminiviridae* | *Begomovirus* | Watermelon curly mottle virus | WCMV | 38 |
| 44 | *Geminiviridae* | *Curtovirus* | Beet curly top virus | BCTV | 39 |
| 45 | *Luteoviridae* | *Polerovirus* | Cucurbit aphid borne yellows virus | CABYV | 40 |
| 46 | *Luteoviridae* | *Polerovirus* | Luffa aphid borne yellows virus | LABYV | 41 |
| 47 | *Luteoviridae* | *Polerovirus* | Melon aphid borne yellows virus | MABYV | 42 |
| 48 | *Luteoviridae* | *Polerovirus* | Pepo aphid borne yellows virus | PABYV | 43 |
| 49 | *Luteoviridae* | *Polerovirus* | Suakwa aphid borne yellows virus | SABYV | 44 |
| 50 | *Partiviridae* | *Unassigned* | Citrullus lanatus cryptic virus | CiLCV | 45 |
| 51 | *Phenuiviridae* | *Unassigned* | Watermelon crinkle leaf associated virus 1 | WCLAV-1 | 46 |
| 52 | *Phenuiviridae* | *Unassigned* | Watermelon crinkle leaf associated virus 2 | WCLAV-2 | 46 |
| 53 | *Potyviridae* | *Ipomovirus* | Cucumber vein yellowing virus | CVYV | 47 |
| 54 | *Potyviridae* | *Ipomovirus* | Squash vein yellowing virus | SVYV | 48 |
| 55 | *Potyviridae* | *Ipomovirus* | Squash yellow leaf curl virus | SYLCV | 49 |
| 56 | *Potyviridae* | *Potyvirus* | Algerian watermelon mosaic virus | AWMV | 50 |
| 57 | *Potyviridae* | *Potyvirus* | Clover yellow vein virus | CYVV | 51 |
| 58 | *Potyviridae* | *Potyvirus* | Cucumber vein banding virus | CVBV | 52 |
| 59 | *Potyviridae* | *Potyvirus* | Moroccan watermelon mosaic virus | MWMV | 53 |
| 60 | *Potyviridae* | *Potyvirus* | Papaya ringspot virus | PRSV | 54 |
| 61 | *Potyviridae* | *Potyvirus* | Soybean mosaic virus | SyMV | 6 |
| 62 | *Potyviridae* | *Potyvirus* | Telfairia mosaic virus | TeMV | 55 |
| 63 | *Potyviridae* | *Potyvirus* | Turnip mosaic virus | TuMV | 56 |
| 64 | *Potyviridae* | *Potyvirus* | Watermelon leaf mottle virus | WLMV | 57 |
| 65 | *Potyviridae* | *Potyvirus* | Watermelon mosaic virus | WMV | 58 |
| 66 | *Potyviridae* | *Potyvirus* | Wild melon vein banding virus | WMVBV | 59 |
| 67 | *Potyviridae* | *Potyvirus* | Zucchini shoestring virus | ZSV | 60 |
| 68 | *Potyviridae* | *Potyvirus* | Zucchini tigre mosaic virus | ZTMV | 61 |
| 69 | *Potyviridae* | *Potyvirus* | Zucchini yellow fleck virus | ZYFV | 62 |
| 70 | *Potyviridae* | *Potyvirus* | Zucchini yellow mosaic virus | ZYMV | 63, 64 |
| 71 | *Rhabdoviridae* | *Nucleorhabdovirus* | Eggplant mottled dwarf virus | EMDV | 65 |
| 72 | *Rhabdoviridae* | *Unassigned* | Cucumber toad skin virus | CTSV | 66 |
| 73 | *Secoviridae* | *Comovirus* | Bean pod mottle virus | BPMV | 6 |
| 74 | *Secoviridae* | *Comovirus* | Squash mosaic virus | SqMV | 67 |
| 75 | *Secoviridae* | *Fabavirus* | Cucurbit mild mosaic virus | CMMV | 68 |
| 76 | *Secoviridae* | *Nepovirus* | Melon mild mottle virus | MMMV | 69 |
| 77 | *Secoviridae* | *Nepovirus* | Tobacco ringspot virus | TRSV | 3 |
| 78 | *Secoviridae* | *Torradovirus* | Squash chlorotic leaf spot virus | SCLSV | 70 |
| 79 | *Tombusviridae* | *Alphanecrovirus* | Tobacco necrosis virus A | TNVA | 3 |
| 80 | *Tombusviridae* | *Aureusvirus* | Cucumber leaf spot virus | CLSV | 71 |
| 81 | *Tombusviridae* | *Carmovirus* | Cucumber soil borne virus | CSBV | 66 |
| 82 | *Tombusviridae* | *Carmovirus* | Melon necrotic spot virus | MNSV | 72 |
| 83 | *Tombusviridae* | *Tombusvirus* | Cucumber bulgarian virus | CBV | 73 |
| 84 | *Tombusviridae* | *Tombusvirus* | Cucumber necrosis virus | CNV | 74 |
| 85 | *Tospoviridae* | *Orthotospovirus* | Watermelon bud necrosis virus | WBNV | 75, 76 |
| 86 | *Tospoviridae* | *Orthotospovirus* | Watermelon silver mottle virus | WSMV | 77 |
| 87 | *Tospoviridae* | *Tospovirus* | Melon yellow spot virus | MYSV | 78 |
| 88 | *Tymoviridae* | *Tymovirus* | Chayote mosaic virus | ChMV | 79 |
| 89 | *Tymoviridae* | *Tymovirus* | Melon roguse mosaic virus | MRMV | 80 |
| 90 | *Tymoviridae* | *Tymovirus* | Squash necrosis virus | SNV | 66 |
| 91 | *Vigaviridae* | *Tobamovirus* | Cucumber green mottle mosaic virus | CGMMV | 81 |
| 92 | *Vigaviridae* | *Tobamovirus* | Cucumber fruit mottle mosaic virus | CFMMV | 82 |
| 93 | *Vigaviridae* | *Tobamovirus* | Cucumber mottle virus | CuMV | 83 |
| 94 | *Vigaviridae* | *Tobamovirus* | Kyuri green mottle virus | KGMV | 81 |
| 95 | *Vigaviridae* | *Tobamovirus* | Tobacco mosaic virus | TMV | 84 |
| 96 | *Vigaviridae* | *Tobamovirus* | Zucchini green mottle mosaic virus | ZGMMV | 85 |

**Table S2.** Complete genomes of CABYV isolates available in GenBank. The US isolate is shown in bold script.

| No. | Country | Host | Isolate | Accession # | Genome (nucleotide) | Reference/s |
| --- | --- | --- | --- | --- | --- | --- |
| 1 | Brazil | Melon | JMB1 | LC217994 | 5737 | 86 |
| 2 | Brazil | Melon | M3 | LC217993 | 5736 | 86 |
| 3 | China | Cantaloupe | Xinjiang | EU636992 | 5682 | Direct submission |
| 4 | China | Cucurbit | Beijing | EU000535 | 5682 | 42 |
| 5 | China | Squash | CABYV-FJ | GQ221223 | 5682 | Direct submission |
| 6 | China | Zucchini | CABYV-CZ | HQ439023 | 5691 | Direct submission |
| 7 | East Timor | Cucumber | 50AL | KY617826 | 5677 | Direct submission |
| 8 | France | Cucurbit | N | NC_003688 | 5669 | 87 |
| 9 | Indonesia | Cucumber | - | LC472499 | 5593 | Direct submission |
| 10 | Japan | Cucumber | CABYV-JAN | GQ221224 | 5682 | Direct submission |
| 11 | Papua New Guinea | Cucumber | 10PN | MG780352 | 5671 | 88 |
| 12 | South Korea | Melon | SW64 | KR231963 | 5681 | 89 |
| 13 | South Korea | Melon | SW25 | KR231962 | 5682 | 89 |
| 14 | South Korea | Melon | SW2 | KR231961 | 5683 | 89 |
| 15 | South Korea | Melon | SW1(14) | KR231960 | 5682 | 89 |
| 16 | South Korea | Melon | SW1 | KR231959 | 5683 | 89 |
| 17 | South Korea | Melon | NW18 | KR231958 | 5683 | 89 |
| 18 | South Korea | Melon | NW5 | KR231957 | 5683 | 89 |
| 19 | South Korea | Melon | NW2(14) | KR231956 | 5683 | 89 |
| 20 | South Korea | Melon | NW2 | KR231955 | 5682 | 89 |
| 21 | South Korea | Melon | NW1 | KR231954 | 5683 | 89 |
| 22 | South Korea | Melon | HS2 | KR231953 | 5682 | 89 |
| 23 | South Korea | Melon | HS1 | KR231952 | 5682 | 89 |
| 24 | South Korea | Melon | HD118 | KR231951 | 5683 | 89 |
| 25 | South Korea | Melon | HD1 | KR231950 | 5680 | 89 |
| 26 | South Korea | Melon | GS6 | KR231949 | 5682 | 89 |
| 27 | South Korea | Melon | GS2 | KR231948 | 5683 | 89 |
| 28 | South Korea | Melon | GS1 | KR231947 | 5682 | 89 |
| 29 | South Korea | Melon | GM16 | KR231946 | 5681 | 89 |
| 30 | South Korea | Melon | GM7 | KR231945 | 5683 | 89 |
| 31 | South Korea | Melon | CY6 | KR231944 | 5682 | 89 |
| 32 | South Korea | Melon | CY4 | KR231943 | 5684 | 89 |
| 33 | South Korea | Melon | CY3 | KR231942 | 5683 | 89 |
| 34 | South Korea | Melon | K1 | LC082306 | 5688 | 90 |
| 35 | South Korea | Watermelon | WM-YS10 | MG257903 | 5683 | Direct Submission |
| 36 | South Korea | Melon | M-CY31 | MG257902 | 5683 | Direct Submission |
| 37 | South Korea | Melon | M-BY1 | MG257901 | 5683 | Direct Submission |
| 38 | South Korea | Cucumber | C-HS1 | MG257900 | 5683 | Direct Submission |
| 39 | South Korea | Cucumber | C-AS1 | MG257899 | 5683 | Direct Submission |
| 40 | Spain | Squash | Sq/2004/1.9 | JF939814 | 5672 | 91 |
| 41 | Spain | Squash | Sq/2005/9.2 | JF939813 | 5675 | 91 |
| 42 | Spain | Squash | Sq/2003/7.2 | JF939812 | 5672 | 91 |
| 43 | Taiwan | Sponge gourd | CABYV-R-TW82 | JQ700306 | 5679 | 92 |
| 44 | Taiwan | Bitter melon | CABYV-C-TW20 | JQ700306 | 5670 | 92 |
| **45** | **USA** | **Pumpkin** | **BL-4** | **MK055337** | **5679** | **93** |

References

1. Fuji, S.; Shinoda, K.; Ikeda, M.; Furuya, H.; Naito, H.; Fukumoto, F. Complete nucleotide sequence of the new potexvirus “Alstroemeria virus X”. *Arch. Virol.;* **2005**,*150*(11), 2377-2385

2. Menzel, W.; Abang, M. M.; Winter, S. Characterization of cucumber vein-clearing virus, a whitefly (Bemisia tabaci G.)-transmitted carlavirus. *Arch. Virol.;* **2011**,*156*(12), 2309-2311.

3. Lovisolo, O. Virus and viroid diseases of cucurbits. In *III Conference on Epidemiology and Control of Virus Diseases of Vegetables.* ***1979****, 88* (pp. 33-82).

4. Gholamalizadeh, R.; Vahdat, A.; Hossein-Nia, S. V.; Elahinia, A.; Bananej, K. Occurrence of Ourmia melon virus in the Guilan Province of Northern Iran. *Plant. Dis*. **2008**, *92*(7), 1135-1135.

5. Xin, M.; Zhang, P.; Liu, W.; Ren, Y.; Cao, M.; Wang, X. The complete nucleotide sequence and genome organization of a novel betaflexivirus infecting Citrullus lanatus. *Arch. Virol.* **2017**, *162*(10), 3239-3242.

6. Ali, A.; Abdalla, O.; Bruton, B.; Fish, W.; Sikora, E.; Zhang, S.; Taylor, M. Occurrence of viruses infecting watermelon, other cucurbits, and weeds in the parts of southern United States. *Plant. Health. Prog.* **2012**,*13*(1), 9.

7. Palukaitis, P.; Roossinck, M. J.; Dietzgen, R. G.; Francki, R. I. Cucumber mosaic virus. In *Advances in virus research*.; Academic Press: 1992,Volume 41, pp. 281-348.

8. Sweet, J. B.; Barbara, D. J. A yellow mosaic disease of horse chestnut (Aesculus spp.) caused by apple mosaic virus. *Annals. Appl. Biol*. **1979**, *92*(3), 335-341.

9. Greber, R. S.; Teakle, D. S.; Mink, G. I. Thrips-facilitated transmission of prune dwarf and prunus necrotic ringspot viruses from cherry pollen to cucumber. *Plant. Dis.* **1992**, *76*(10), 1039-1041.

10. Thien, H. X.; Bhat, A. I.; Jain, R. K. Mungbean necrosis disease caused by a strain of Groundnut bud necrosis virus. *Indian. Phytopathol*. **2003**, *56*(1), 54-60.

11. Ciuffo, M.; Kurowski, C.; Vivoda, E.; Copes, B.; Masenga, V.; Falk, B. W.; Turina, M. A new Tospovirus sp. in cucurbit crops in Mexico. *Plant. Dis.* **2009**, *93*(5), 467-474.

12. Bezerra, I. C.; de O. Resende, R.; Pozzer, L.; Nagata, T.; Kormelink, R.; De Avila, A. C. Increase of tospoviral diversity in Brazil with the identification of two new tospovirus species, one from chrysanthemum and one from zucchini. *Phytopathology.* **1999**, *89*(9), 823-830.

13. Rubio, L.; Soong, J.; Kao, J.; Falk, B. W. Geographic distribution and molecular variation of isolates of three whitefly-borne closteroviruses of cucurbits: Lettuce infectious yellows virus, Cucurbit yellow stunting disorder virus, and Beet pseudo-yellows virus. *Phytopathology*. **1999**, *89*(8), 707-711.

14. Hartono, S.; Natsuaki, T.; Genda, Y.; Okuda, S. Nucleotide sequence and genome organization of Cucumber yellows virus, a member of the genus Crinivirus. *J. Gen. Virol.* **2003**, *84*(4), 1007-1012.

15. Okuda, M.; Okazaki, S.; Yamasaki, S.; Okuda, S.; Sugiyama, M. Host range and complete genome sequence of Cucurbit chlorotic yellows virus, a new member of the genus Crinivirus. *Phytopathology*. **2010**, *100*(6), 560-566.

16. Duffus, J. E.; Larsen, R. C.; Liu, H. Y. Lettuce infectious yellows virus-a new type of whitefly-transmitted virus. *Phytopathology*. **1986**, *76*(1), 97-100.

17. Sabanadzovic, S.; Wintermantel, W. M.; Valverde, R. A.; McCreight, J. D.; Aboughanem-Sabanadzovic, N. Cucumis melo endornavirus: genome organization, host range and co-divergence with the host. *Virus. Res.* **2016***,* *214*, 49-58.

18. Kwon, S. J.; Tan, S. H.; Vidalakis, G. (2014). Complete nucleotide sequence and genome organization of an endornavirus from bottle gourd (Lagenaria siceraria) in California, USA. *Virus. Genes*. **2014**, *49*(1), 163-168.

19. Peng, X.; Pan, H.; Muhammad, A.; An, H.; Fang, S.; Li, W.; Zhang, S. Complete genome sequence of a new strain of Lagenaria siceraria endornavirus from China. *Arch. Virol.* **2018***, 163*(3), 805-808.

20. Guzman, P.; Sudarshana, M. R.; Seo, Y. S.; Rojas, M. R.; Natwick, E.; Turini, T.; Gilbertson, R. L. A new bipartite geminivirus (begomovirus) causing leaf curl and crumpling in cucurbits in the Imperial Valley of California. *Plant. Dis.* **2000**, *84*(4), 488-488.

21. Brown, J. K.; Idris, A. M.; Alteri, C.; Stenger, D. C. (2002). Emergence of a new cucurbit-infecting begomovirus species capable of forming viable reassortants with related viruses in the Squash leaf curl virus cluster. *Phytopathology*. **2002**, *92*(7), 734-742.

22. Tahir, M.; Haider, M. S.; Briddon, R. W. Complete nucleotide sequences of a distinct bipartite begomovirus, bitter gourd yellow vein virus, infecting Momordica charantia. *Arch. Virol.* **2010**, *155*(11), 1901-1905.

23. Mandal, B.; Mandal, S.; Sohrab, S. S.; Pun, K. B.; Varma, A. A new yellow mosaic disease of chayote in India. *Plant. Pathol.* **2004**, *53*(6).

24. Revill, P. A.; Ha, C. V.; Porchun, S. C.; Vu, M. T.; Dale, J. L. The complete nucleotide sequence of two distinct geminiviruses infecting cucurbits in Vietnam. *Arch. Virol.* **2003**,*148*(8), 1523-1541.

25. Brown, J. K.; Idris, A. M.; Rogan, D.; Hussein, M. H.; Palmieri, M. Melon chlorotic leaf curl virus, a new begomovirus associated with Bemisia tabaci infestations in Guatemala. *Plant. Dis.* **2001**, *85*(9), 1027

26. Romay, G.; Chirinos, D.; Geraud-Pouey, F.; Desbiez, C. Association of an atypical alphasatellite with a bipartite New World begomovirus. *Arch. Virol.* **2010**, *155*(11), 1843-1847.

27. Muniyappa, V.; Maruthi, M. N.; Babitha, C. R.; Colvin, J.; Briddon, R. W.; Rangaswamy, K. T. Characterisation of pumpkin yellow vein mosaic virus from India. *Annals. Appl. Biol.* **2003**,*142*(3), 323-331.

28. Hong, Y.; Wang, X.; Tian, B.; Cai, J. Chinese squash leaf curl virus: a new whitefly-transmitted geminivirus. ***Sci. China, Ser. B, Chem. Life Sci. Earth Sci.*** **1995**, *38*(2), 179-186.

29. Kon, T.; Dolores, L. M.; Bajet, N. B.; Hase, S.; Takahashi, H.; Ikegami, M. Molecular characterization of a strain of Squash leaf curl China virus from the Philippines. *J. Phytopathol.* **2003**, *151*(10), 535-539.

30. Cohen, S.; Duffus, J. E.; Larsen, R. C.; Liu, H. Y.; Flock, R. A. Purification, serology, and vector relationships of squash leaf curl virus, a whitefly-transmitted geminivirus. *Phytopathology*. **1983**, *73*(12), 1669-1673.

31. Xie, Y.; Zhou, X. P. Molecular characterization of squash leaf curl Yunnan virus, a new begomovirus and evidence for recombination. *Arch. Virol.* **2003**, 148(10), 2047-2054.

32. Castro, R. M.; Moreira, L.; Rojas, M. R.; Gilbertson, R. L.; Hernández, E.; Mora, F.; Ramírez, P. Occurrence of Squash yellow mild mottle virus and Pepper golden mosaic virus in Potential New Hosts in Costa Rica. *Plant. Pathol. J.* **2013**, *29*(3), 285.

33. Leke, W. N.; Khatabi, B.; Fondong, V. N.; Brown, J. K. Complete genome sequence of a new bipartite begomovirus infecting fluted pumpkin (Telfairia occidentalis) plants in Cameroon. *Arch. Virol. 2016, 161*(8), 2347-2350.

34. Jones, D. R. Plant viruses transmitted by whiteflies. *Euro. J. Plant. Pathol.* **2003**, *109*(3), 195-219.

35. Juárez, M.; Tovar, R.; Fiallo-Olivé, E.; Aranda, M. A.; Gosálvez, B.; Castillo, P.; Navas-Castillo, J. First detection of Tomato leaf curl New Delhi virus infecting zucchini in Spain. *Plant. Dis.* **2014**, *98*(6), 857-857.

36. Zubiaur, Y. M.; Fonseca, D.; Quiñones, M.; Palenzuela, I. Presence of Tomato yellow leaf curl virus infecting squash (Curcubita pepo) in Cuba. *Plant. Dis.* **2003**, *88*(5), 572-572.

37. Jones, P.; Sattar, M. H. A.;Al Kaff, N. incidence of virus disease in watermelon and sweetmelon crops in the Peoples Democratic Republic of Yemen and its impact on cropping policy. *Asp. Appl.* Biol. **1988**, 203-207.

38. Brown, J. K.; NELSON, M. R. Characterisation of watermelon curly mottle virus, a geminivirus distinct from squash leaf curl virus. *Annals. Appl. Biol.* **1989**, *115*(2), 243-252.

39. Wang, H.; de A. Gurusinghe, P.; Falk, B. W.Systemic insecticides and plant age affect beet curly top virus transmission to selected host plants. *Plant. Dis.* **1999**, *83*(4), 351-355.

40. Lecoq, H.; Bourdin, D.; WIPF‐SCHEIBEL, C.; Bon, M.; Lot, H.; Lemaire, O.; Herrbach, E. A new yellowing disease of cucurbits caused by a luteovirus, cucurbit aphid‐borne yellows virus. *Plant. Pathol.* **1992**, *41*(6), 749-761.

41. Knierim, D.; Tsai, W. S.; Maiss, E.; Kenyon, L. Molecular diversity of poleroviruses infecting cucurbit crops in four countries reveals the presence of members of six distinct species. *Arch. Virol.* **2014**,*159*(6), 1459-1465.

42. Xiang, H. Y.; Shang, Q. X.; Han, C. G.; Li, D. W.; Yu, J. L. Complete sequence analysis reveals two distinct poleroviruses infecting cucurbits in China. *Arch. Virol.* **2008**,*153*(6), 1155.

43. Ibaba, J. D.; Laing, M. D.; Gubba, A. Pepo aphid-borne yellows virus: a new species in the genus Polerovirus. *Virus. Genes*. **2017**, *53*(1), 134-136.

44. Shang, Q. X.; Xiang, H. Y.; Han, C. G.; Li, D. W.; Yu, J. L. Distribution and molecular diversity of three cucurbit-infecting poleroviruses in China. *Virus. Res.* **2009**,*145*(2), 341-346.

45. Sela, N.; Lachman, O.; Reingold, V.; Dombrovsky, A. A new cryptic virus belonging to the family Partitiviridae was found in watermelon co-infected with Melon necrotic spot virus. *Virus. Genes*. **2013**, *47*(2), 382-384.

46. Xin, M.; Cao, M.; Liu, W.; Ren, Y.; Zhou, X.; Wang, X. Two negative-strand RNA viruses identified in watermelon represent a novel clade in the order Bunyavirales. *Front. Microbiol.* **2017**, *8*, 1514.

47. Lecoq, H.; Desbiez, C.; Delécolle, B.; Cohen, S.; Mansour, A. Cytological and molecular evidence that the whitefly-transmitted Cucumber vein yellowing virus is a tentative member of the family Potyviridae The GenBank accession number of the sequence reported in this paper is AF233429. *J. Gen. Virol.* **2000**, *81*(9), 2289-2293.

48. Egel, D. S.; Adkins, S. Squash vein yellowing virus identified in watermelon (Citrullus lanatus) in Indiana. *Plant. Dis.* **2007**, *91*(8), 1056-1056.

49. Zouba, A. A.; Lopez, M. V.; Anger, H. Squash yellow leaf curl virus: a new whitefly-transmitted poty-like virus. *Plant. Dis.* **1998**, *82*(5), 475-478.

50. Yakoubi, S.; Lecoq, H.; Desbiez, C. Algerian watermelon mosaic virus (AWMV): a new potyvirus species in the PRSV cluster. *Virus. Genes*. **2008**, *37*(1), 103-109.

51. Inouye, N.; Maeda, T.; Mitsuhata, K. A strain of clover yellow vein virus isolated from Calanthe sp. In *VII International Symposium on Virus Diseases of Ornamental Plants*. 1988, *234* (pp. 61-68).

52. Perotto, M. C.; Pozzi, E. A.; Celli, M. G.; Luciani, C. E.; Mitidieri, M. S.; Conci, V. C. Identification and characterization of a new potyvirus infecting cucurbits. *Arch. Virol.* **2018**, *163*(3), 719-724.

53. McKern, N. M.; Strike, P. M.; Barnett, O. W.; Ward, C. W.; Shukla, D. D. Watermelon mosaic virus-Morocco is a distinct potyvirus. *Arch. Virol.* **1993**, *131*(3-4), 467-473.

54. Purcifull, D. E.; Hiebert, E. Serological distinction of watermelon mosaic virus isolates. *Phytopathology*. **1979**, *69*(2), 112-116.

55. Shoyinka, S. A.; Brunt, A. A.; Phillips, S.; Lesemann, D. E.; Thottappilly, G.; Lastra, R. The occurrence, properties and affinities of Telfairia mosaic virus, a potyvirus prevalent in Telfairia occidentalis (Cucurbitaceae) in South Western Nigeria. *J. Phytopathol.* **1987**, 1, 13-24.

56. Lecoq, H.; Desbiez, C. Viruses of cucurbit crops in the Mediterranean region: an ever-changing picture. In *Advances in virus research*.; Academic Press: Cambridge, MA, USA, 2012, Volume 84, pp. 67-126.

57. De Sa, P. B.; Hiebert, E.; Purcifull, D. E. Molecular characterization and coat protein serology of watermelon leaf mottle virus (Potyvirus). *Arch. Virol.* **2000**,*145*(3), 641-650.

58. Webb, R. E.; Scott, H. A. Isolation and identification of Watermelon mosaic viruses 1 and 2. *Phytopathology*. **1965**, *55*(8), 895-900.

59. Desbiez, C.; Wipf-Scheibel, C.; Millot, P.; Verdin, E.; Dafalla, G.; Lecoq, H. New species in the papaya ringspot virus cluster: Insights into the evolution of the PRSV lineage. *Virus. Res.* **2017**, *241*, 88-94.

60. Ibaba, J. D.; Laing, M. D.; Gubba, A. Zucchini shoestring virus: a distinct potyvirus in the papaya ringspot virus cluster. *Arch. Virol.* **2016**, *161*(8), 2321-2323.

61. Romay, G.; Lecoq, H.; Desbiez, C. Zucchini tigré mosaic virus is a distinct potyvirus in the papaya ringspot virus cluster: molecular and biological insights. *Arch. Virol.* **2014**, *159*(2), 277-289.

62. Vovlas, C.; Hiebert, E.; Russo, M. Zucchini yellow fleck virus, a new potyvirus of zucchini squash. *Phytopathol. Mediterr.* **1981**, 123-128.

63. Lisa, V.; Boccardo, G.; D'Agostino, G.; Dellavalle, G.; d'Aquilio, M. Characterization of a potyvirus that causes zucchini yellow mosaic. *Phytopathology*. **1981**, *71*(7), 667-672.

64. Lecoq, H.; Pitrat, M.; Clément, M. Identification et caractérisation d’un potyvirus provoquant la maladie du rabougrissement jaune du melon. *Agronomie*. **1981**, *1*(10), 827-834.

65. Roggero, P.; Milne, R. G.; Masenga, V.; Ogliara, P.; Stravato, V. M. First reports of eggplant mottled dwarf rhabdovirus in cucumber and in pepper. *Plant. Dis.* **1995**, *79*(3).

66. Lecoq, H.; Wisler, G.; Pitrat, M. Cucurbit viruses: the classics and the emerging. In *Cucurbitaceae*. 1992, Volume 98, pp. 126-142.

67. Freitag, J. H. Beetle Transmission, Host Range, and Properties of Squash Mosaic Virus. *Phytopathology*. **1956**, *46*(2).

68. Dong, S. W.; Xiang, H. Y.; Shang, Q. X.; Li, D. W.; Yu, J. L.; Han, C. G. Complete genomic sequence analysis reveals a novel fabavirus infecting cucurbits in China. *Arch. Virol.* **2012**, *157*(3), 597-600.

69. Tomitaka, Y.; Usugi, T.; Yasuda, F.; Okayama, H.; Tsuda, S. A novel member of the genus Nepovirus isolated from Cucumis melo in Japan. *Phytopathology*. **2011**, *101*(3), 316-322.

70. Lecoq, H.; Verdin, E.; Tepfer, M.; Wipf-Scheibel, C.; Millot, P.; Dafalla, G.; Desbiez, C. Characterization and occurrence of squash chlorotic leaf spot virus, a tentative new torradovirus infecting cucurbits in Sudan. *Arch. Virol.* **2016**, (6), 1651-1655.

71. Miller, J. S.; Damude, H.; Robbins, M. A.; Reade, R. D.; Rochon, D. M. Genome structure of cucumber leaf spot virus: sequence analysis suggests it belongs to a distinct species within the Tombusviridae. *Virus. Res.* 1997, *52*(1), 51-60.

72. Riviere, C. J.; Rochon, D. M. Nucleotide sequence and genomic organization of melon necrotic spot virus. *J. Gen. Virol.* **1990**, *71*(9), 1887-1896.

73. Kostova, D.; Lisa, V.; Rubino, L.; Marzachì, C.; Roggero, P.; Russo, M. Properties of cucumber Bulgarian latent virus, a new species in the genus Tombusvirus. *J. Plant. Pathol.* **2003**, 85 (1) 27-33.

74. McKeen, C. D. Cucumber necrosis virus. *Can. J. Bot.* **1959**, *37*(5), 913-925.

75. Jain, R. K.; Pappu, H. R.; Pappu, S. S.; Reddy, M. K.; Vani, A. Watermelon bud necrosis tospovirus is a distinct virus species belonging to serogroup IV. *Arch. Virol.* **1998**, *143*(8), 1637-1644.

76. Singh, S. J.; Krishnareddy, M. Watermelon bud necrosis: a new tospovirus disease. In *Tospoviruses and Thrips of Floral and Vegetable Crops*. 1995, *431*, 68-77.

77. Yeh, S. D.; Chang, T. F. Nucleotide sequence of the N gene of watermelon silver mottle virus, a proposed new member of the genus Tospovirus. *Phytopathology*. **1995**, *85*(1), 58-64.

78. Kato, K.; Handa, K.; Kameya-Iwaki, M. Melon yellow spot virus: a distinct species of the genus Tospovirus isolated from melon. *Phytopathology*. **2000**, *90*(4), 422-426.

79. Bernal, J. J.; Jiménez, I.; Moreno, M.; Hord, M.; Rivera, C.; Koenig, R.; Rodríguez-Cerezo, E. Chayote mosaic virus, a new tymovirus infecting Cucurbitaceae. *Phytopathology*. **2000**, *90*(10), 1098-1104.

80. Jones, P.; Angood, S. B.; Carpenter, J. M. Melon rugose mosaic virus, the cause of a disease of watermelon and sweet melon. *Annals. Appl. Biol.* **1986**, *108*(2), 303-307.

81. Francki, R. I.; Hu, J.; Palukaitis, P. Taxonomy of cucurbit-infecting tobamoviruses as determined by serological and molecular hybridization analyses. *Intervirol.* **1986**, *26*(3), 156-163.

82. Antignus, Y.; Wang, Y.; Pearlsman, M.; Lachman, O.; Lavi, N.; Gal-On, A. Biological and molecular characterization of a new cucurbit-infecting tobamovirus. *Phytopathology*. **2001**, *91*(6), 565-571.

83. Orita, H.; Sakai, J. I.; Kubota, K.; Okuda, M.; Tanaka, Y.; Hanada, K.; Iwanami, T. Molecular and serological characterization of cucumber mottle virus, a new cucurbit-infecting tobamo-like virus. *Plant. Dis.* **2007**, *91*(12), 1574-1578.

84. Webb, R. E.; Foster, R. E. A strain of tobacco mosaic virus isolated from muskmelon. *Pl. Dis. Reptr*. **1966**, *50*, 49-52.

85. Ryu, K. H.; Min, B. E.; Choi, G. S.; Choi, S. H.; Kwon, S. B.; Noh, G. M.; Cho, K. H. Zucchini green mottle mosaic virus is a new tobamovirus; comparison of its coat protein gene with that of kyuri green mottle mosaic virus. *Arch. Virol.* **2000**, *145*(11), 2325-2333.

86. Costa, T. M.; Blawid, R.; Aranda, M. A.; Freitas, D. M. S.; Andrade, G. P.; Inoue-Nagata, A. K.; Nagata, T. Cucurbit aphid-borne yellows virus from melon plants in Brazil is an interspecific recombinant. *Arch. Virol.* **2019**, *164*(1), 249-254.

87. Guilley, H.; Wipf-Scheibel, C.; Richards, K.; Lecoq, H.; Jonard, G. Nucleotide sequence of cucurbit aphid-borne yellows luteovirus. *Virology*. **1994**, *202*(2), 1012-1017.

88. Maina, S.; Barbetti, M. J.; Edwards, O. R.; Minemba, D.; Areke, M. W.; Jones, R. A. First complete genome sequence of Cucurbit aphid-borne yellows virus from Papua New Guinea. *Genome.* Announc. **2018**, *6*(11).

89. Kwak, H. R.; Lee, H. J.; Kim, E. A.; Seo, J. K.; Kim, C. S.; Lee, S. G.; Kim, M. Complete Genome Sequences and Evolutionary Analysis of Cucurbit aphid-borne yellows virus Isolates from Melon in Korea. *Plant. Pathol. J*. **2018**, *34*(6), 532.

90. Choi, S. K.; Yoon, J. Y.; Choi, G. S. (2015). Biological and molecular characterization of a Korean isolate of Cucurbit aphid-borne yellows virus infecting Cucumis species in Korea. *Plant. Pathol. J*. **2015**, *31*(4), 371.

91. Kassem, M. A.; Juarez, M.; Gómez, P.; Mengual, C. M.; Sempere, R. N.; Plaza, M.; Aranda, M. A. Genetic diversity and potential vectors and reservoirs of Cucurbit aphid-borne yellows virus in southeastern Spain. *Phytopathology*. **2013**, *103*(11), 1188-1197.

92. Knierim, D.; Tsai, W. S.; Deng, T. C.; Green, S. K.; & Kenyon, L. Full‐length genome sequences of four polerovirus isolates infecting cucurbits in Taiwan determined from total RNA extracted from field samples. *Plant. Pathol.* **2013**, *62*(3), 633-641.

93. Khanal, V.; & Ali, A. First Complete Genome Sequence of Cucurbit Aphid-Borne Yellows Virus from Pumpkin in the United States. *Microbiol. Resour. Announc.* **2019**, *8*(6).
