# Supplementary figures and images for: High Prevalence of Three Potyviruses Infecting Cucurbits in Oklahoma and Phylogenetic Analysis of Cucurbit Aphid-Borne Yellows Virus Isolated from Pumpkins
Source: Pathogens. 2021 Jan 8;10(1):53. doi: 10.3390/pathogens10010053 (PMC7828045; doi:10.3390/pathogens10010053)

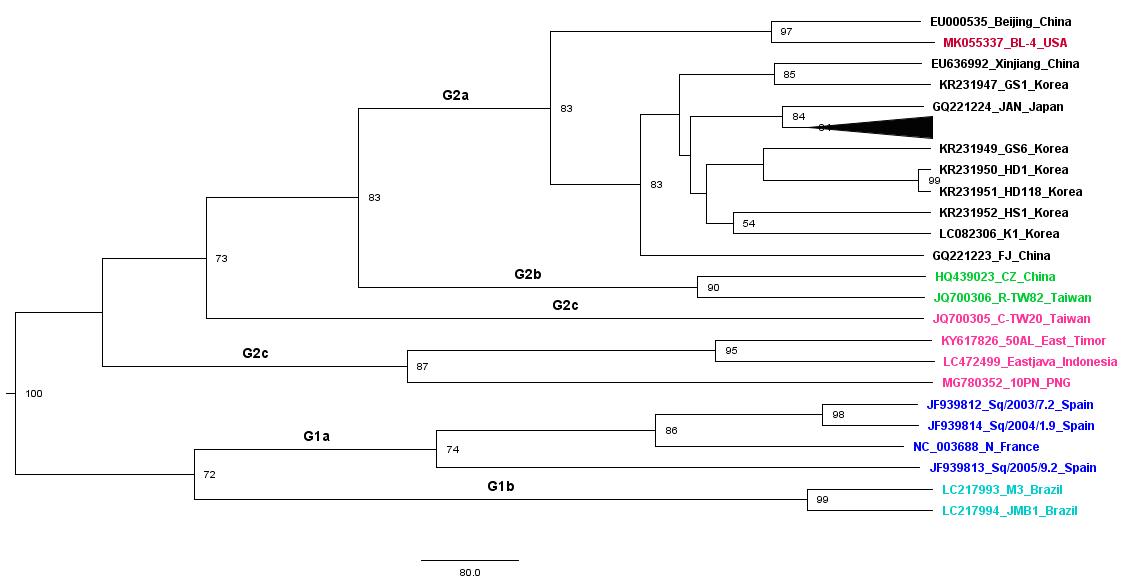

Supplement: Supplementary file 1 [file pathogens-10-00053-s001.zip › Figure S1.revised.tif]
